# Constitutive Activation of STAT3 Signaling Regulates hTERT and Promotes Stem Cell-Like Traits in Human Breast Cancer Cells

**DOI:** 10.1371/journal.pone.0083971

**Published:** 2013-12-30

**Authors:** Seyung S. Chung, Clement Aroh, Jaydutt V. Vadgama

**Affiliations:** 1 Division of Cancer Research and Training, Department of Internal Medicine, Charles R. Drew University of Medicine and Science, Los Angeles, California, United States of America; 2 Jonsson Comprehensive Cancer Center, David Geffen UCLA School of Medicine, Los Angeles, California, United States of America; University of Newcastle, United Kingdom

## Abstract

Mounting clinical data suggest that high telomerase activity is tightly associated with cancer progression and poor outcomes. Constitutively activated STAT3 is found in ∼60% of human malignancies and shows a dismal prognosis. We previously reported that activated STAT3 promoted epithelial-mesenchymal transition (EMT) and cancer stem cell phenotype in human breast cancer. However, little is known how STAT3 is regulated in the cancer stem cell and by which mechanisms STAT3 contributes to poor prognosis in aggressive breast cancer. Here we demonstrate that STAT3 physically interacts with CD44 and NF-kB and activates the catalytic subunit of telomerase (hTERT) in human breast cancer stem cells. STAT3 plays a role as a signal transducing molecule between CD44 and NF-kB. In addition to functioning as a catalytic subunit of telomerase, hTERT has been reported to function as a transcription co-factor which drives EMT and cancer stem cell phenotype in human cancer. We observed that activated hTERT increases CD44 (+) subpopulation, whereas targeted knock-down of hTERT abolished cancer stem cell phenotype. Targeted STAT3 knock-down cells also down-regulated hTERT and decreased CD44 subpopulation. Finally, CD44 knock-down resulted in the abrogation of cancer stem cell phenotype and concurrent down-regulation of pSTAT3 and hTERT. Our study delineates the signaling pathway where STAT3 functions as a modulator for CD44 and hTERT, promoting a cancer stem cell phenotype. The constitutive activation of STAT3 signaling that leads to regulation of hTERT pathway may provide novel therapeutic targets for human breast cancer stem cells.

## Introduction

Worldwide, breast cancer is the most common malignancy in women accounting for 22.9% of all cancers [1]. Although much progress has been made in breast cancer treatment modalities and improvement of patient survival and quality of life, the patients with breast cancer continue to die of the diseases [2]. Increasing evidences suggested that tumors possess a heterogeneous population of cells in which specific subgroup of cells are chemo-resistant, radio-resistant, promoting tumor recurrence and metastasis [3]. This subpopulation of cancer cells are denoted cancer stem cells (CSCs). For many cancers, including breast cancer, the tumorigenesis is initiated and sustained by the cancer stem cells [4]. The ineffectiveness of current cancer therapy has been indicated to reflect the lack of activity against CSCs which remain viable despite therapy. Therefore, it is critical to identify the signaling pathways selectively activated in the CSCs in order to target them. Pharmacological targeting cancer stem cells might be an excellent modality for the breast cancer treatment.

Transcription factors found constitutively activated in CSCs include STAT3 and NF-kB [5], [6]. Signal transducer and activator of transcription 3 (STAT3) is a latent cytoplasmic transcription factor that conveys various signals of cytokines and growth factors from the cell membrane to nucleus [7]. In a variety of human malignancies, including breast cancer, constitutive activation of STAT3 is correlated with the tumor progression and a poor prognosis [8]. Recent studies with human breast and lung cancer tissues demonstrated that an activated STAT3 is the crucial contributor to invasion and migration [9]–[10]. STAT3 is activated through tyrosine phosphorylation (pSTAT3) by a variety of cytokines, implicating that it integrates diverse signals into common transcriptional response [11]. However, the molecular mechanisms by which STAT3 is promoting cancer stem cell traits in breast cancer, as well as the potential contributions of STAT3 to metastasis, have yet to be defined.

NF-kB transcription factor has been observed to be constitutively activated in many human cancers [12]. NF-kB has been demonstrated to contribute to cancer cell proliferation, survival, metastasis and therapeutic resistance as well as regulation of genes involved in immunity and inflammation [13]–[14]. Notably, NF-kB blocks apoptosis by stimulating anti-apoptotic genes and suppressing apoptosis inducing genes [15].

Human telomerase reverse transcriptase (hTERT) is a catalytic component of telomerase, RNA-dependent DNA polymerase that elongates telomeric DNA [16]. Recent studies have revealed the level of hTERT expression is closely correlated with a clinical aggressiveness and poor prognosis in many human malignancies [17]–[20]. Telomerase and hTERT expressions are activated up to 90% of human malignancies as targeting telomerase or hTERT structure has been suggested for the cancer therapy [21]. In addition to its requirement in telomeric extension, hTERT has been implicated for multiple essential roles for oncogenesis [22]–[24]. Ectopic expression of hTERT was shown to promote malignant transformation independently of telomere lengthening [25]. Recently, hTERT was shown to stimulate EMT and induce stemness in human gastric cancer cells, thereby promote cancer metastasis and recurrence [26].

Here we report that pSTAT3 activates hTERT and promotes cancer stem cell phenotype in breast cancer. We report that STAT3 binds NF-kB to activate hTERT. Then, the activated hTERT stimulates the cancer stem cell marker CD44 expression and enhances the invasiveness and migration. CD44 interacts with STAT3 and convey the signals to hTERT signaling pathway. Our study suggests STAT3-NF-kB-hTERT signaling axis may provide a novel target-therapy for cancer progression and metastasis in human breast tumors.

## Methods

### Ethics Statement

N/A.

### Cancer Cell Lines and Culture

BT474, SKBR3, MDA-MB-231 and MCF7 wild type cell lines were obtained from the American Type Culture Collection (ATCC). They were maintained in a monolayer culture in DMEM/F12 (Dulbecco’s modified Eagle medium) with 10% fetal bovine serum, 2.5% L-Glutamine and 0.5% Penicillin/Streptomycin. The MCF7-HER2 and MCF7-neo (MCF7 cells transfected with HER2 plasmid and a control vector, respectively) cell lines [27] were generous gift of Dr. C. Kent Osborne (Baylor College of Medicine, Houston, Texas). MCF7-HER2 and MCF7-neo cells were maintained in a monolayer culture in DMEM 1x with 10% fetal bovine serum, 2.5% L-Glutamine, 0.5% Penicillin/Streptomycin, and G418 (400 µg/ml). All of our experiments involving MCF7 were carried out with both MCF7 and MCF7/neo cells. Because similar results were obtained from both cell lines, only results for MCF7 are reported in this work.

### Tumorosphere Formation Assay

Matrigel (BD, Cambridge, MA), 200 µl was spread as a thick layer on a 24 well plate and allowed to polymerize at 37°C for 15 minutes. 2×10^4^ Cells grown as monolayer were trypsinized to single cells and plated on top of the pre-coated Matrigel. Plates were incubated at 37°C to allow cells to fully settle down before media was replaced with appropriate culture media containing 5% Matrigel. Cells were grown for 15 days; fresh growth media with Matrigel was replenished every two days. Images of representative fields were taken.

### Boyden-Chamber Invasion Assay

Mouse Fibroblasts (NIH-3T3) were used as a chemo attractant, and grown in a 24- well plate in 2 ml of the same media as MCF7-HER2 cells. MCF7 WT, MCF7-HER2 and STAT3 knock-down experimental cells were synchronized to an equal number (125,000 cells) in a 6- well plate and were serum starved overnight. Boyden chambers were prepared with 25 µl of 1∶6 diluted Matrigel and allowed to incubate for about 2 hours to solidify. After cell synchronization, invasion was allowed to occur for 40 hours. The cells were then fixed with 0.5% glutaraldehyde and stained with 5% toluidine blue for cell counting. Three different microscope fields of 40x were used to quantify the invasion statistics when counting cells.

### Short Hairpin Interfering RNA Transfection

The hTERT, STAT3, CD44 and HER2 short hairpin RNAs and control shRNA were purchased from the Ori Gene (Rockville, MD). The transfection was performed by using lipofectamine 2000 (Invitrogen) reagents following the manufacturer’s instructions. The HER2 and STAT3 shRNA knock- down expressions after shRNA transfection were determined by a Western blot analysis at 48 to 72 hours of transfection.

### Real-Time Quantitative PCR

Complementary DNA (cDNA) was synthesized by reverse transcription (RT) with Thermoscript™ RT-PCR system (Invitrogen, Carlsbad, CA) according to the manufacturer’s instructions. Q-PCR analysis was carried out according to manufacturer's instructions (Invitrogen) on a Q-PCR I-Cycler machine (Bio-Rad). 18S was used as an internal control. In the analysis, relative breast cancer cell line gene expression was compared to MCF-7 WT gene expression by setting MCF-7 WT expression to “1”. Then, for example, MCF-7-HER2 and MDA-MB-231 gene expression were compared based up on this MCF-7 WT's set expression level through “folds” of increase or decrease. Primer sequences used are as follows:

hTERT Forward: 5′-CCG TCT GCG TGA GGA GAT-3′ Reverse: 5′-TGG GGA TGA AGC GGA GTC-3′ 18S Forward: 5′-GATCCATTGGAGGGCAAGTC-3′, Reverse: 5′-TCCCAAGATCCAACTACGAG-3′.. Annealing temperature used was 58 degrees Celsius unless otherwise noted.

### Western Blot Analyses

Monolayer cultures of respective cell lines at an 80–90% confluence were lysed using 100 µl of RIPA buffer (Thomas Scientific Inc). Tris-glycine (Bio-Rad) gels were loaded with 50–100 µg of lysates. After running gel electrophoresis, the gel was transferred to a nitrocellulose membrane for 2 hours. The membrane was blocked for 1 hour in 5% BSA or 5% skim milk at 4°C. The membrane was then washed 3 times with 1xTTBS and incubated overnight with the primary antibody at 4°C. Human TERT primary antibody was purchased from Millipore (MABE 14, Billerica, MA). Primary antibodies of Oct-4, Sox-2, STAT3, pSTAT3, E-cadherin, Vimentin, slug and β-actin were purchased from Cell Signaling Technology (Danvers, MA).

### Immunoprecipitation

Cells were washed once with PBS buffer and lysed in immunoprecipitation lysis buffer (Thomas Scientific Company). Antibodies to STAT3, NF-kB (Cell signaling technology) and hTERT (R&D systems) were added to the cell lysates and incubated for one hour at 4°C. Protein A agarose (Santa cruz biotechnology) were added to the cell lysates 70 µl each, incubated for overnight at 4°C. After the immunoprecipitation, cell lysates were briefly spun and washed three times with PBS buffer. Immuno-pellets were re-suspended in 100 µl of loading dye (Bio Rad) and resolved on a 7.5% polyacrylamide gel electrophoresis. For cytoplasmic and nuclear extract preparation, nuclear and cytoplasmic extraction kit (Thomas Scientific, catalog number 78833) was used following the manufacturer’s instructions.

### Chromatin Immunoprecipitation Assay

Chromatin immunoprecipitation (Chip) Assay Kit (Millipore, Catalog number 17–295) was utilized to study STAT3 binding to hTERT promoter region. MCF7-HER2 cells or MDA-MB-231 cells were incubated with 1% formaldehyde for 20 minutes at 37°C. Cells were collected, lysed, sonicated, and incubated with 4 µg of antibodies to STAT3 overnight. PCR was used to amplify DNA bound to the immunoprecipitated histones after reversing the histone-DNA cross-links. Primer sets were designed flanking the possible STAT3 binding regions. Primer sequences: *hTERT* promoter primer sequence 1, forward primer 5′-CCAAACCTGTGGACAGAACC-3′ and reverse primer 5′-AGACTGACTGCCTCCATCGT-3′ and hTERT promoter primer sequence 2, forward primer 5′-GGGGTGTCTTCTGGGTATCA-3′ and reverse primer 5′-AAGGGCTGTGTTTGTGAATTG-3′.

### FACS Profile Analysis

Approximately 500,000 cells of MDA-MB-231 or MCF7-HER2 cells were washed with 1xPBS, trypsinized and then transferred to a 15 ml aliquot tube. Cell suspensions were centrifuged down, re-suspended in 2 ml 1xPBS and then divided into two tubes 0.5 ml each for staining. One tube was used as an unstained control and the other one as stained with 10 µl CD44 Antibody (FITC Green; BD Biotech). The tubes were vortexed briefly and incubated at room temperature for 15 minutes in the dark. Each tube was then washed with 3.5 ml 1xPBS and then centrifuged down for 6 minutes. After aspirating the supernatant, the cells were re-suspended in 3 ml 1xPBS and subjected to the FACS profiling at UCLA FACS Core laboratory.

## Results

### STAT3 Physically Binds CD44 and NF-kB and hTERT Expression was Up-regulated in STAT3 Activated Breast Cancer Cell Lines

To define molecular function of STAT3 in breast cancer, we chose two breast cancer cell lines which contain the constitutively activated STAT3 (pSTAT3), MDA-MD-231 and MCF7-HER2. In MDA-MB-231, STAT3 was activated through IL-6/JAK2 signaling [28] whereas in MCF7-HER2, STAT3 was activated by the HER2 overexpression [29]. MDA-MB-231 is a triple-negative, ER, PR and HER2 lacking cell line and is also an invasive cancer cell line. MCF7 wild-type does not possess CD44 (+) subpopulation whereas MCF7-HER2 and MDA-MB-231 both express high CD44 (+) subpopulation. To uncover the molecular partners of STAT3, we performed the immunoprecipitation assay with the STAT3 monoclonal antibody. Once we obtained the immuno-pellets, we ran a western blot to find which proteins are bound to STAT3.

**Figure 1:**
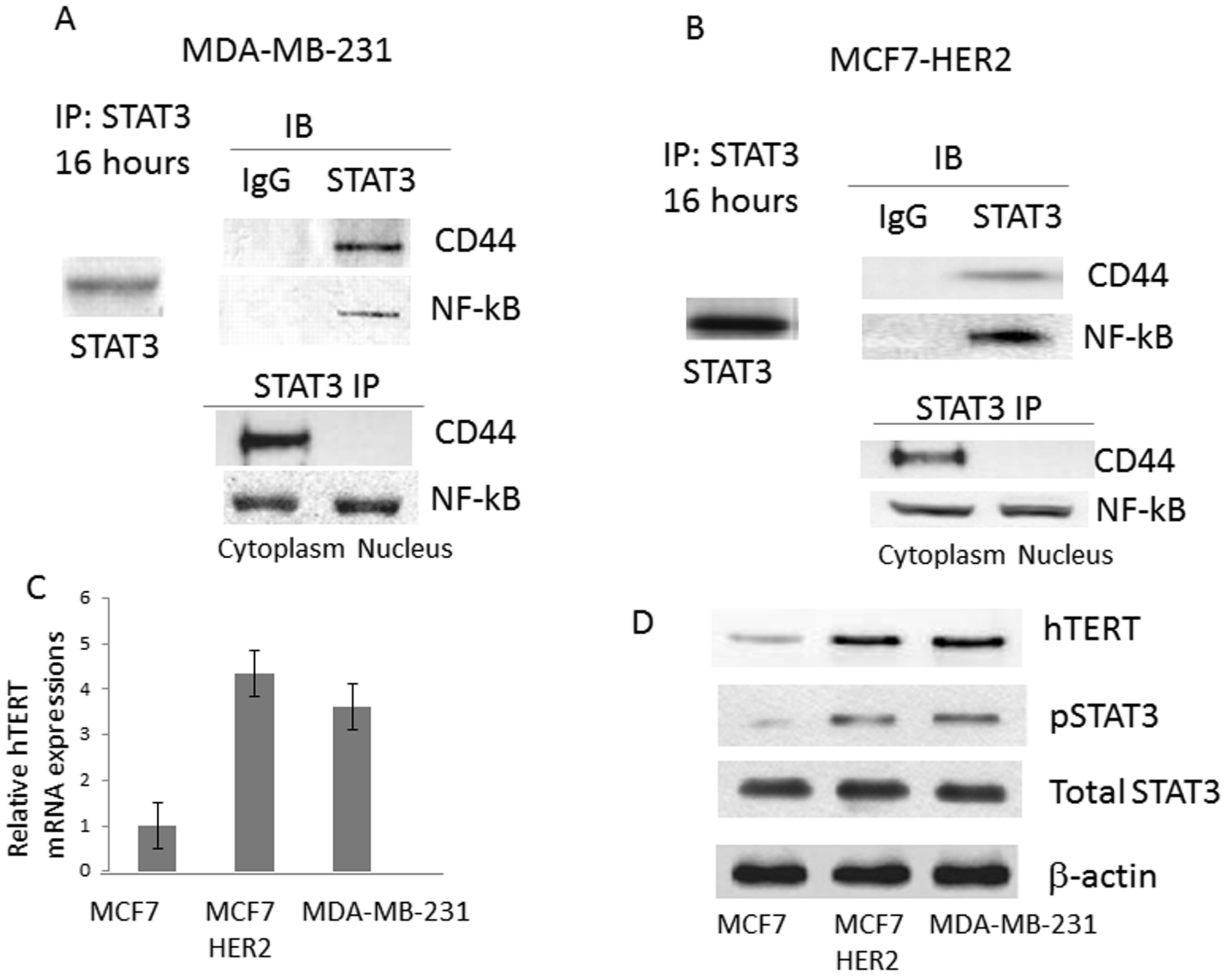
STAT3 binds NF-kB and CD44 in breast cancer and telomerase reverse transcriptase (hTERT) is up-regulated in pSTAT3 activated breast cancer cells. A: Immuno-precipitation with STAT3 antibody revealed that NF-kB and CD44 were bound to STAT3 in MDA-MB-231 cell line. STAT3 pull-down was performed in the cytoplasm and nuclear extracts separately. STAT3 was bound CD44 in the cytoplasm and bound to NF-kB in both cytoplasm and nuclear extracts. B: Pull-down assay with STAT3 antibody showed that STAT3 interaction with NF-kB and CD44 held true in MCF7-HER2. C: Real time PCR data showed that hTERT mRNA expressions were up-regulated in pSTAT3 activated cancer cell lines. MCF7 wild type was used as a control for hTERT mRNA expression of 1 fold. D: Western blot analyses: Up-regulation of hTERT expression was associated with pSTAT3 activation in human breast cancer.

As shown in Figure 1, STAT3 was bound to both CD44 and NF-kB in MDA-MB-231 and MCF7-HER2 cell lines (Figure 1A and B). CD44, a transmembrane glycoprotein, is a major receptor for extracellular proteins involved in invasion and metastasis of human cancers [30]. Therefore, CD44 has been recognized as one of the key cell surface biomarkers for cancer stem cells in breast cancer [31]. NF-kB is a nuclear factor that functions in cancer cell proliferation, survival and metastasis. STAT3 and NF-kB both are involved in the gene regulation of inflammation. When we pulled down STAT3 in the cytoplasm and nucleus extracts separately, we found that STAT3 bound CD44 in the cytoplasm but not in the nucleus (Figure 1A and 1B). However, STAT3 was bound to NF-kB both in the cytoplasm and nucleus. Our data suggest that STAT3 is the linking molecule that connects CD44 and NF-kB signaling in aggressive breast cancer cell lines.

Next we determined the gene expression levels of human telomerase reverse transcriptase (hTERT) in the breast cancer cell lines. Konnikova and associates have demonstrated that STAT3 regulated the expression of hTERT in human glioblastoma and primary cells [32]. Since we noticed that STAT3 binds NF-kB from the pull-down assay, we wanted to determine if hTERT expression has been changed in pSTAT3 activated cancer cells. To test this, we performed the RT-PCR and western blot for the hTERT in MCF7-HER2 and MDA-MB-231 cell lines. RNA expression was increased 4.3 folds in MCF7-HER2 compared to MCF7 wild type and also increased 3.6 folds in MDA-MB-231, respectively (Figure 1C). To monitor hTERT protein expression level, western blot was performed. In agreement with the PCR data, hTERT protein expression was up-regulated in both MCF7-HER2 and MDA-MB-231 (Figure 1D). Total STAT3 expression levels were comparable among three cell lines, however pSTAT3 was expressed in MDA-MB-231 and MCF7-HER2 only. This pSTAT3 expression was concurrent with hTERT up-regulation.

### Transcription Factor STAT3 Binds STAT3 Sites within the Promoter Region of hTERT Gene

**Figure 2:**
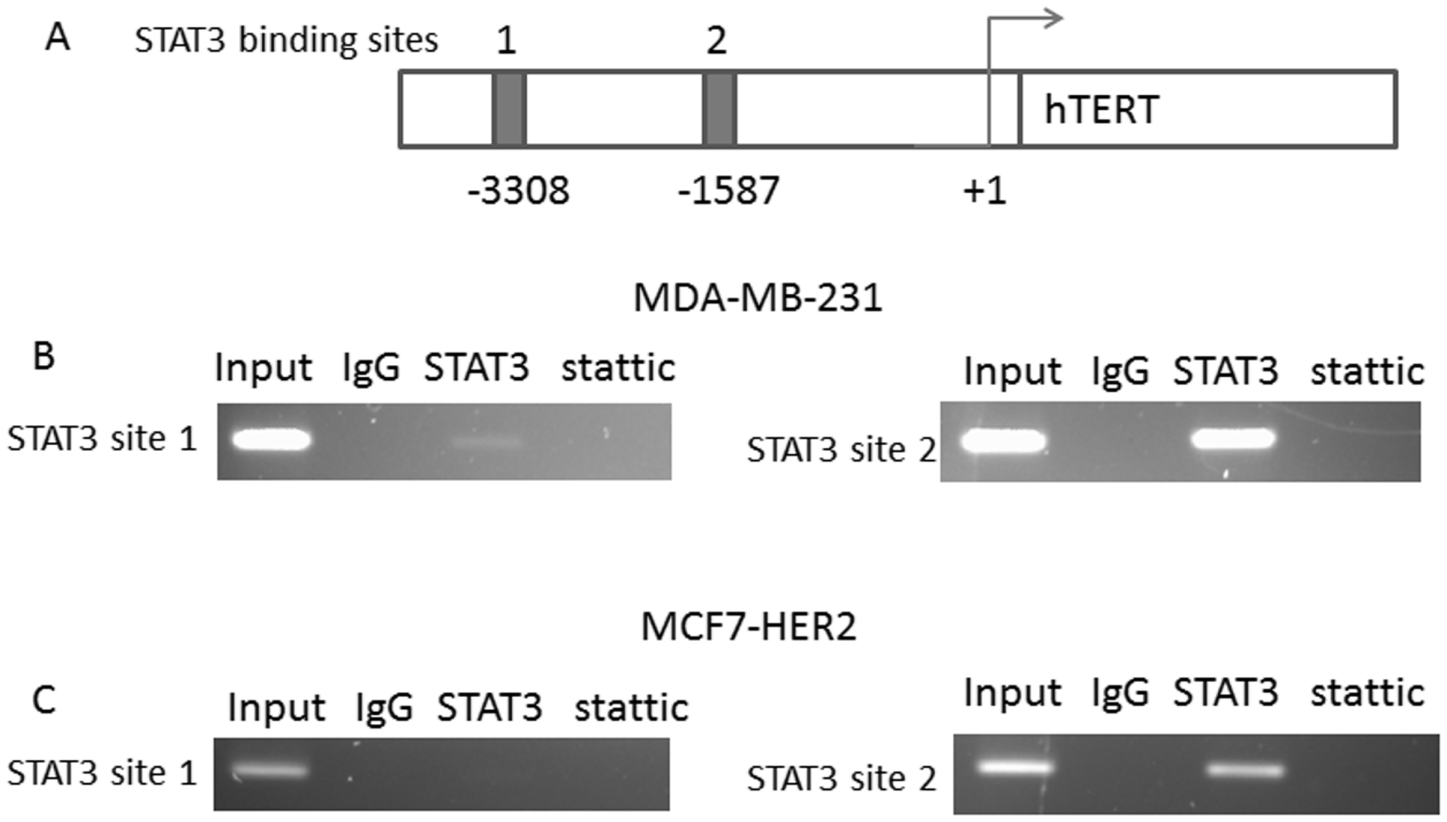
STAT3 binds to the hTERT promoter region. The promoter region of the **hTERT** gene possessed consensus STAT3-binding sites. Primer sets were designed flanking the putative STAT3-binding sites 1 and 2. Chip assay was performed with the primers. **A:** Diagram of hTERT promoter with location of consensus STAT3-binding sites 1 and 2 indicated. B: Chip assay was done on the MDA-MB-231 lysates using anti-STAT3 antibody or IgG (control) and protein-A agarose beads as described in Materials and Methods. STAT3 inhibitor stattic was treated for 24 hours and subjected to Chip assay to monitor STAT3 binding to hTERT promoter. Input samples are DNAs amplified from lysates before immunoprecipitation. C: Chip assay was done on the MCF7-HER2 lysates using anti-STAT3 antibody or IgG (control) and protein-A agarose beads.

The findings that constitutively activated STAT3 upregulates hTERT expression suggest that STAT3 may directly regulate the hTERT gene. We wished to determine whether transcription factor STAT3 binds the promoter region of hTERT gene. It was shown that consensus STAT3-binding sites (TTCNNNGAA) reside within the hTERT promoter [32]. Chromatin immunoprecipitation (Chip) assays were done with two putative STAT3-binding sites (Figure 2A). In MDA-MB-231 cells, we found STAT3 bound to STAT3 binding site 1 weakly and to bind site 2 more strongly in the hTERT promoter (Figure 2B). STAT3 can be found bound to STAT3 binding site 2 of the hTERT promoter, but not to STAT3 binding site 2 in MCF7-HER2 (Figure 2C). Chemical inhibitor of STAT3, stattic, treatment for 24 hours led to an abolishment of STAT3 binding to the STAT3 sites 1 and 2 (Figure 2B and 2C). Our Chip data are consistent with the RT-PCR and western data that show an increase of hTERT expression with STAT3 activation. These results suggest that STAT3 directly regulates hTERT expression in aggressive breast cancer cell lines.

### Targeted Knock-down of STAT3 and/or Specific Inhibition of STAT3 Down-regulated hTERT and CD44 Expression Levels

**Figure 3:**
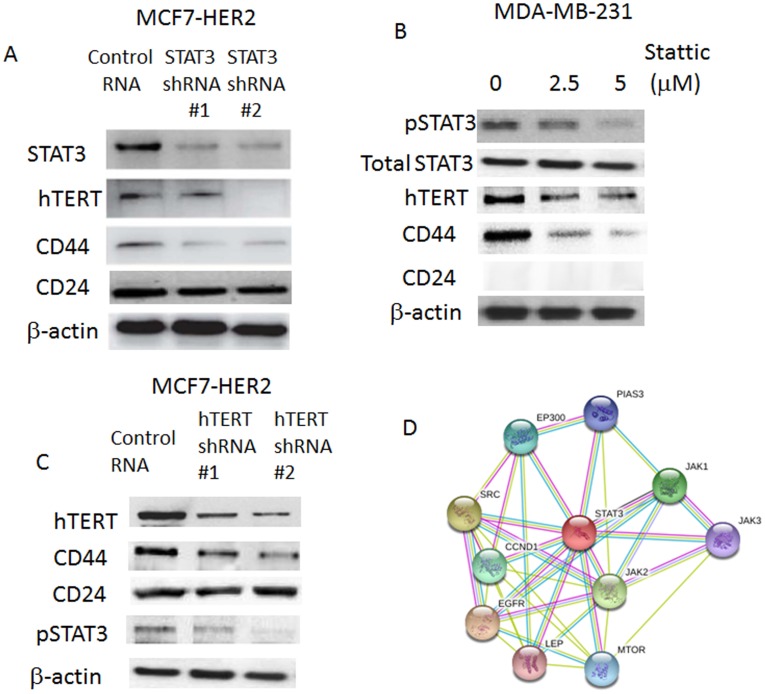
Inhibition of pSTAT3 down-regulated hTERT as well as hTERT knock-down down-regulated CD44. Targeted knock-down of STAT3 and chemical inhibition of pSTAT3 both down-regulated hTERT and CD44 expression levels. A: STAT3 shRNA mediated knock-down cells showed decreased expression levels of both hTERT and CD44. Western blot analyses were performed with STAT3 knock-down cells for monitoring hTERT, CD44 and CD24. B: Chemical inhibition of pSTAT3 with stattic also showed the reduced expression levels of hTERT and CE44. Stattic (5 µM) was treated for 24 hours, then proteins expression levels of pSTAT3, hTERT, CD44 and CD24 were examined. C: Western analyses of hTERT knock-down also revealed that CD44 reduction and pSTAT3 inhibition. D: Schematic representation of the interaction of the STAT3 protein with the STRING analysis. Protein-protein interaction database STRING was utilized to search the STAT3 protein interactions.

Since hTERT expression levels were up-regulated in the pSTAT3 activated breast cancer cell lines, we next wished to determine whether STAT3 is the genuine cellular factor that regulates hTERT. To prove this, we took two experimental approaches. One is to transcriptionally repress STAT3 and examine hTERT expression. The other approach is to specifically inhibit STAT3 phosphorylation by the chemical inhibitor, stattic. After we transfected MCF7-HER2 with shRNA specific for STAT3, we monitored the hTERT and CD44 expression levels (Figure 3). As shown in this figure, when STAT3 expression was knocked-down, both hTERT and CD44 expression levels were clearly decreased. This indicates that STAT3 is required for the hTERT gene expression in the breast cancer cells.

In another independent approach, we blocked the STAT3 phosphorylation step and observed the hTERT and CD44 protein expression levels. To this end, MDA-MB-231 cells were treated with the STAT3 chemical inhibitor, stattic, and western blot was performed to confirm the hTERT and CD44 protein expression. Again, consistent with shRNA knock-down, hTERT was down-regulated when STAT3 phosphorylation was inhibited at the post-translational step. Our data suggest that STAT3 activation is responsible for the hTERT up-regulation in the aggressive breast cancer cell lines.

Next, we determined the hTERT’s role in the pSTAT3 and CD44 expression in the same cell lines. To this end, we knocked-down the hTERT and examined the pSTAT3 and CD44 expression levels (Figure 3C). As shown in this figure, when hTERT was knocked-down, both CD44 and pSTAT3 expression levels were down-regulated. These data suggest that hTERT regulates CD44 and STAT3 activation in an integrated manner in the breast cancer cell lines. This is important in that hTERT can control the cancer stem cell marker, CD44, and pSTAT3 which physically bind to CD44 in the integrated signal pathway. We searched the string protein-protein interaction database for STAT3 networks (Figure 3D). STAT3 interactions with CD44 and NF-kB are novel ones in the system. CD44-STAT3 regulation on to hTERT expression is also new signaling axis based on the data set.

### The Targeted Knock-down of hTERT Suppressed the Cell Invasiveness of Breast Cancer Cells

**Figure 4:**
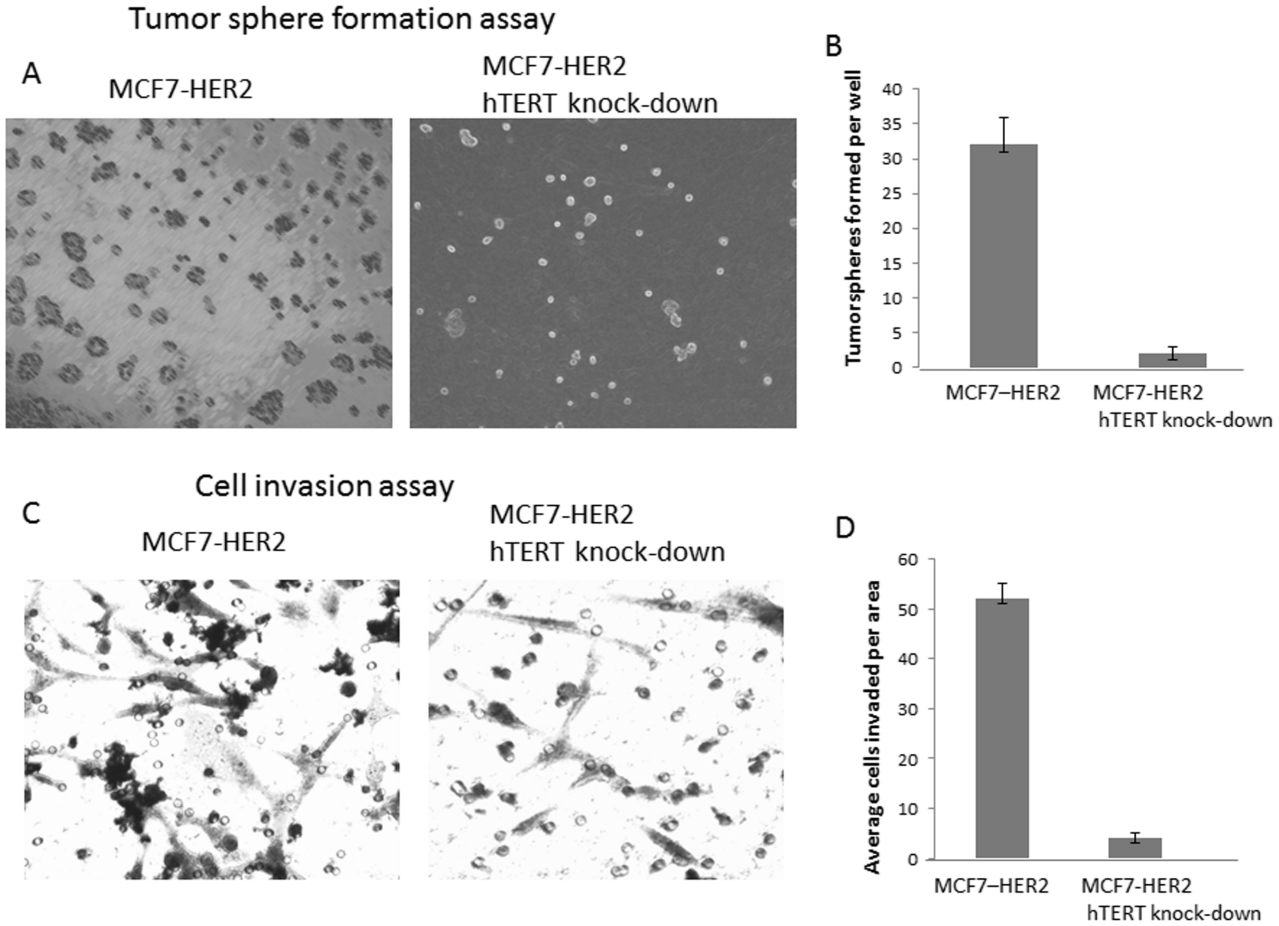
Transcriptional silencing of hTERT suppressed the tumorigenecity and invasiveness *in vitro*. A: Tumorosphere formation assay. When hTERT was knocked-down, the tumorosphere formation was inhibited *in vitro*. hTERT knock-down cells were subjected to three dimensional culture condition and were examined for tumor sphere formation after 5 days. B: Quantitative representation of tumorospheres formed in MCF7-HER2 and hTERT knock-down cells. C: Boyden chamber assay. Cell invasiveness was examined by employing Boyden chamber assay. MCF7-HER2 and hTERT knock-down cells were subjected to Boyden chamber assay. Again, less cells invaded the filters when hTERT expression was silenced. D: The cell invasion assay was quantitatively measured in graphic representation.

As hTERT shRNA-mediated gene silencing down-regulated cancer stem cell marker CD44 and inhibited STAT3 phosphorylation, we next examined invasiveness properties of the hTERT knocked-down cells. To this end, we employed two cell invasion assays; tumorosphere formation and Boyden chamber assay. In tumorosphere culture condition, we created three-dimensional microenvironment by adding 5% matrigel into the 24 well plates. Each well, we seeded 20,000 cells and cultured until the tumorospheres were formed. As shown in the Figure 4, MCF7-HER2 transfected with control RNA formed tumorospheres in 5 days. However, when we knocked-down the hTERT from the same cell line, the tumorosphere forming capacity has been significantly reduced (Figure 4 A and B). When hTERT gene was knocked-down, the tumorosphere formation number has been decreased ∼10 folds in the MCF7-HER2. In agreement with the tumorosphere culture, cell invasion assay also revealed that hTERT knock-down has inhibited the cell invasiveness (Figure 4C and D). The average cells invaded from the Boyden chamber has been decreased from 52 to 4 cells when compared MCF7-HER2 control RNA and MCF7-HER2 hTERT shRNA transfected cells. Our results suggest that hTERT is contributing the cancer stem cell phenotype including the cell invasiveness.

### MCF7-HER2 and MDA-MB-231 Possess High CD44 (+) Subpopulation

**Figure 5:**
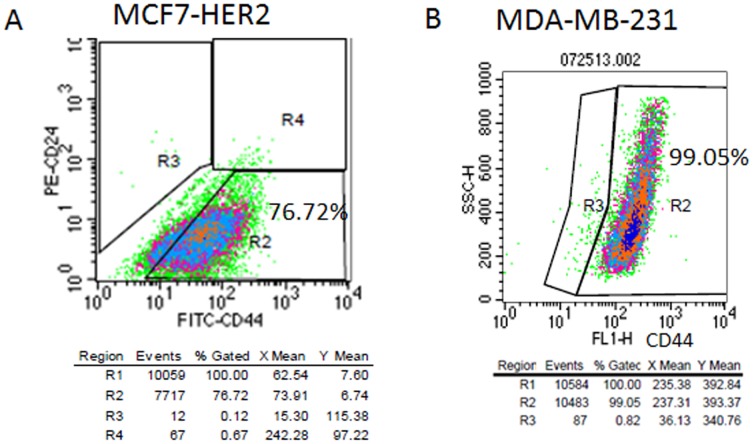
Both MCF7-HER2 and MDA-MB-231 possess high CD44 (+) cellular populations. A: FACS profiles of MCF7_HER2. CD44-FITC was stained for MCF7-HER2 (passage 30) and subjected to FACS profiling. As shown in the figure, MCF7-HER2 possessed 76.72% of CD44 (+) cell population. B. CD44 FACS profiles of MDA-MB-231. CD44-FITC was stained for MDA-MB-231 and subjected FACS profiling. MDA-MB-231 possessed 99.05% of CD44 (+) cell population.

The cancer stem cell criteria that we chose were CD44 positivity, tumor sphere formation and boyden chamber invasion. MCF7-HER2 cell lines as well as HER2 positive breast tumor tissues showed the high CD24 positive cell population. Therefore we took CD44 positivity as a cancer stem cell marker and confirmed the tumor sphere formation, cell invasion to define cancer stem cell phenotype. MCF7-HER2, we increased the passage number (∼passage 30) to obtain high CD44(+) cell populations. We found that MCF7-HER2 high passage cells possess 76.72% CD44 (+) (Figure 5A). Likewise, MDA-MB-231 possesses high CD44 (+) of 99.05% (Figure 5B). MCF7-HER2 and MDA-MB-231 CD44 (+) profiles are presented in figure 5.

### CD44 Contributes to the Cancer Stem Cell Phenotype by Regulating pSTAT3 and hTERT, Possibly through an Integrated Signaling Manner

**Figure 6:**
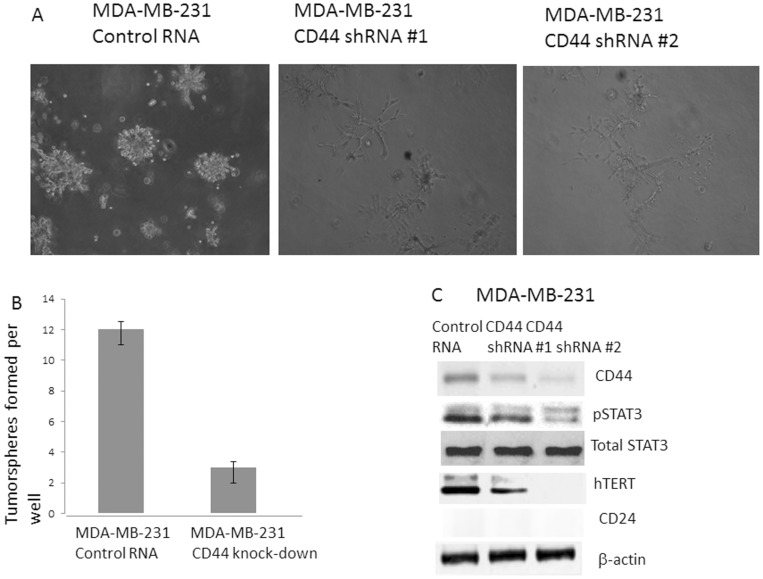
Transcriptional repression of CD44 suppressed the tumorigenecity and pSTAT3 activation. A: Tumorosphere formation assay of CD44 knock-down cells. A representative area was pictured from the tumorosphere cultures from MDA-MB-231 control RNA and CD44 transfected cells. B: Quantitative graph was presented for the tumor sphere formation assay of CD44 knock-down cells. C: Western analyses of CD44 shRNA transfected cells. Protein expression levels were examined for hTERT, pSTAT3, CD44 and CD24 from the CD44 knock-down cells. Downstream genes of pSTAT and hTERT levels were presented.

Since both STAT3 and hTERT transcriptional silencing resulted in the decreased CD44 protein expression (Figure 3). We hypothesized that CD44 might contribute to the cancer stem cell traits by regulating pSTAT3 and hTERT in the integrated signal pathway. We reasoned that once STAT3 up-regulated hTERT, hTERT then started to function as an activator for CD44 in the integrated fashion which triggers the pSTAT3 signaling again, resulting in the cancer stem cell traits. To test this, we first knocked-down CD44 gene from the high CD44 (+) breast cancer cell line MDA-MB-231. Then, we examined the cancer stem cell phenotype and key protein expression in the CD44 knock-down cells. When CD44 gene was knocked-down from MDA-MB-231, the tumorosphere formation has been reduced from ∼12 to 3 spheres in the matrigel microenvironment (Figure 6A and B). Considering CD44 is the key biomarker for cancer stem cell, it is reasonable to expect the reduction in tumorigenecity. To find out the actual molecular events accompanied by the CD44 knock-down, we examined the pSTAT3, total STAT3 and hTERT expression levels. As expected, pSTAT3 activation was reduced, thus hTERT was down-regulated with the CD44 targeted knock-down (Figure 6C). Our results suggest the integrated signaling in that CD44 interact with STAT3, STAT3 gets phosphorylated and translocated into the nucleus, then pSTAT3 binds NF-kB and activates hTERT and hTERT, in return, activates the CD44 expression in a feedback fashion in the constitutively activated STAT3 cancer cells.

## Discussion

Increasing evidence support the concept that many types of malignancies, including breast cancer, originate from cancer stem cell (CSC) population which is able to initiate and spread tumors. Cancer stem cell population represents only small portion in the tumor, however it pertains high capacity to initiate tumors, resist the chemo and radio-therapy and metastasize to other organs. Considering the CSC traits, it is imperative to identify the signal pathways specifically activated in CSCs so that we can devise the strategies to target them.

**Figure 7:**
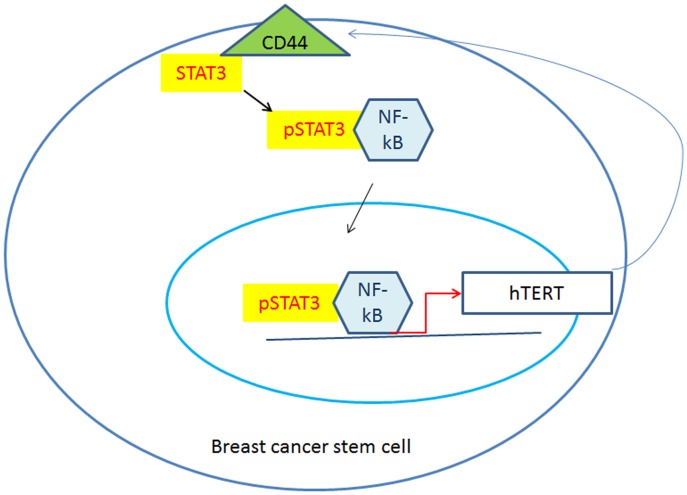
Schematic diagrams of STAT3-hTERT-CD44 autocrine signaling in breast cancer cells. STAT3 was found to bound CD44 and NF-kB concurrently. STAT3-NF-kB complex translocates into nucleus and binds to hTERT promoter and activated hTERT expression. Activated hTERT enhances CD44 expression in an autocrine manner in breast cancer stem cells.

In this report, we describe the novel relationship between CSC and hTERT pathway which involves constitutive activation of STAT3 and NF-kB signaling. STAT3 protein was immuno-precipitated with monoclonal antibody and the subsequent immuno-blotting revealed the binding to NF-kB and CD44 proteins. Examination of protein expression levels in these aggressive cancer cells revealed that pSTAT3 activation is associated with hTERT up-regulation. Targeted knock-down of STAT3 or chemical inhibition of STAT3 phosphorylation both showed the down-regulation of hTERT and CD44. Chip assay revealed that STAT3 binds to consequence sequences of STAT3 binding sites within hTERT promoter in aggressive breast cancer cell lines. This is consistent with previous study which also showed STAT3 binds to the promoter region of hTERT in human glioblastoma [32]. Transcriptional silencing of hTERT suppressed the in vitro tumorigenesis in the tumorosphere formation and Boyden chamber assays. Furthermore, CD44 knock-down inhibited pSTAT3 and down-regulated hTERT. As a result, CD44 knock-down cell showed abolished tumorigenesis in vitro. Taken together, our findings demonstrate that constitutive activation of STAT3 elicits the hTERT signaling which, in turn, up-regulates CD44 in an integrated mode. Our model for cancer stem cell phenotype has been summarized in figure 7. This report provides a valuable model for studying CSC traits in human breast tumors.

The STAT3 signal pathway has been linked to cancer, and it triggers critical target genes regulating cell proliferation and survival. It has been reported that aberrant STAT3 activation promotes uncontrolled tumor cell growth and survival through multiple mechanisms, including increased expression of oncogenes, such as c-myc, Skp2, and cyclin D1, as well as antiapoptotic proteins, including Bcl-2, Bcl-xL, Mcl-1, and survivin [33]–[35]. STAT3 activation is not only essential for cell survival but also required for cell cycle transition [36]. Importantly, STAT3 is now known to regulate growth and self-renewal of glioblastoma stem cells [37]. Therefore, interfering with the STAT3 oncogenic pathway might provide a novel targeted-therapy based on its capability for the CSC population maintenance.

Recent study demonstrated that human telomerase reverse transcriptase (hTERT) can function as a transcription co-factor for the Wnt signaling in embryonic stem cells [38]. Artandi and colleagues have shown that hTERT is specifically functioning as a component of transcriptional complex and up-regulates the target genes of Wnt signaling in stem cells. It opens up new area of non-telomeric function of hTERT in the stem cell and also possibly in cancer cells. In gastric cancer, hTERT was shown to contribute to the epithelial-mesenchymal transition and cancer stem cell traits recently [26]. We have illustrated here that STAT3 phosphorylation up-regulated hTERT then, activated hTERT enhanced cancer stem cell marker CD44 expression in breast cancer. This could explain why constitutive activation of STAT3 is contributing to the cancer stem cell phenotype.

In addition, we defined the molecular cross-talk between STAT3, hTERT and CD44 signaling pathways in aggressive breast cancer cells. Targeted knock-down of STAT3 down-regulated hTERT and CD44, hTERT knock-down reduced CD44 and pSTAT3 expression and finally, CD44 silencing resulted in inhibition of STAT3 phosphorylation and concurrent hTERT downregulation. CD44, a transmembrane glycoprotein, is a major receptor for extracellular proteins, including hyaluronan, involved in invasion and metastasis of human cancers. Many studies relate the CD44 overexpression to the invasive and metastatic phenotype, and indicator for poor prognosis [30]–[31]. Since CD44 does not function as an intrinsic kinase, how it conveys activated signals from numerous growth factors or cytokines is likely through the interactions with other partner kinases, such as receptor tyrosine kinases or intracellular kinases. Therefore, identifying the molecular partners including kinase for CD44 is essential to understand the biological roles of CD44. Our study clearly showed that CD44 interacts with STAT3 then possibly transduces the signals through STAT3-hTERT pathway, which may activate Wnt signaling in the cancer stem cell population. Notably, it was demonstrated that CD44 positive breast cancer stem cells had preferential activation of STAT3, suggesting STAT3 as a potential therapeutic target for human breast tumors [5]. Our data on STAT3 activation are consistent with previous studies on the preferential activation of STAT3 in CD44 positive breast cancer stem cells.

It has to yet to be defined the underlying mechanisms how TERT inhibition blocks pStat3. However, our data reveal that TERT inhibition downregulates CD44, which binds STAT3 physically. Furthermore, CD44 knockdown reduces pStat3 levels, indicating that CD44 promotes Stat3 phosphorylation. Taken together, we propose that activated hTERT increases CD44 expression levels, and CD44 promotes STAT3 phosphorylation by binding Stat3. This may explain why TERT inhibition blocks pSTAT3.

How CD44 knockdown contributes to decline in TERT levels? We have shown that CD44 binds Stat3 and promotes Stat3 phosphorylation. Our Chip data revealed that STAT3 binds to STAT3 sites of hTERT promoter, thereby activates hTERT transcriptionally. In accordance, STAT3 knockdown reduced the TERT expression levels in this work. Our results suggest that CD44-STAT3-hTERT signaling axis exists in cancer stem cell population.

We hypothesize the interactions between CD44, STAT3 and hTERT signaling pathways that occur in the human malignancies are deregulated in the cancer stem cells. As shown in this study, there is a cross-talk between these signaling pathways in the breast cancer stem cells that drive tumorigenesis and metastasis. The constitutive activation of STAT3 and NF-kB and up-regulation of hTERT signaling pathway identifies novel therapeutic targets for breast cancer. Future studies are warranted toward validating these studies *in vivo* and developing targeted-therapy with specific inhibitors for them.
